# Osteogenic Differentiation of Human Gingival Fibroblasts Inhibits Osteoclast Formation

**DOI:** 10.3390/cells13131090

**Published:** 2024-06-24

**Authors:** Merve Ceylan, Ton Schoenmaker, Jolanda M. A. Hogervorst, Ineke D. C. Jansen, Irene M. Schimmel, Caya M. Prins, Marja L. Laine, Teun J. de Vries

**Affiliations:** 1 Department of Periodontology, Academic Centre for Dentistry Amsterdam, University of Amsterdam and Vrije University Amsterdam, Gustav Mahlerlaan 3004, 1081 LA Amsterdam, The Netherlands; 2 Department of Oral Cell Biology, Academic Centre for Dentistry Amsterdam, University of Amsterdam and Vrije University Amsterdam, Gustav Mahlerlaan 3004, 1081 LA Amsterdam, The Netherlands; 3 Department of Medical Biology, Amsterdam University Medical Centers, Location AMC, University of Amsterdam and Vrije University Amsterdam, Gustav Mahlerlaan 3004, 1081 LA Amsterdam, The Netherlands

**Keywords:** gingival fibroblasts, osteoclasts, osteoblasts, osteogenesis, osteoclastogenesis

## Abstract

Gingival fibroblasts (GFs) can differentiate into osteoblast-like cells and induce osteoclast precursors to differentiate into osteoclasts. As it is unclear whether these two processes influence each other, we investigated how osteogenic differentiation of GFs affects their osteoclast-inducing capacity. To establish step-wise mineralization, GFs were cultured in four groups for 3 weeks, without or with osteogenic medium for the final 1, 2, or all 3 weeks. The mineralization was assessed by ALP activity, calcium concentration, scanning electron microscopy (SEM), Alizarin Red staining, and quantitative PCR (qPCR). To induce osteoclast differentiation, these cultures were then co-cultured for a further 3 weeks with peripheral blood mononuclear cells (PBMCs) containing osteoclast precursors. Osteoclast formation was assessed at different timepoints with qPCR, enzyme-linked immunosorbent assay (ELISA), TRAcP activity, and staining. ALP activity and calcium concentration increased significantly over time. As confirmed with the Alizarin Red staining, SEM images showed that the mineralization process occurred over time. Osteoclast numbers decreased in the GF cultures that had undergone osteogenesis. TNF-α secretion, a costimulatory molecule for osteoclast differentiation, was highest in the control group. GFs can differentiate into osteoblast-like cells and their degree of differentiation reduces their osteoclast-inducing capacity, indicating that, with appropriate stimulation, GFs could be used in regenerative periodontal treatments.

## 1. Introduction

Alveolar bone resorption, an important hallmark of periodontitis, occurs as a result of prolonged periodontal inflammation that leads to disturbed balance in bone metabolism in favor of osteoclastogenesis [1]. During chronic inflammation, pro-inflammatory cytokines such as interleukin (IL)-1β, IL-6, and tumor necrosis factor-α (TNF-α) interfere with the balance between bone formation and bone resorption, and cause uncoupling of these two processes. Thus, prolonged inflammation not only induces osteoclast formation and activity but also alters osteoblast function. This has been shown in animal studies where the bone formation phase is affected due to an extended inflammatory process in diabetic animals after an injection of *Porphyromonas gingivalis* (*P. gingivalis*) or the application of cytokines such as IL-1β and TNF-α in vivo [2,3]. This delicate balance in bone metabolism is regulated mostly by the receptor activator of nuclear factor-κB ligand (RANKL) secreted by osteocytes and osteoblasts, by its receptor RANK on osteoclast precursor cells, and by decoy receptor osteoprotegerin (OPG) [4]. This RANKL/RANK/OPG axis is also modulated by other resident cells in the periodontium, such as gingival and periodontal ligament fibroblasts [5].

Gingival fibroblasts (GFs) are amongst the most abundant cell types in the gingival tissue, where they regulate tissue maintenance and integrity by producing extracellular matrix (ECM) proteins, mainly type I collagen. They also modulate immune and inflammatory responses by regulating cell adhesion of leukocytes, generating high amounts of reactive oxygen species, and inducing proliferation of T-cells [6,7,8]. Under inflammatory status, GFs release several bacteria-induced inflammatory mediators like IL-6, IL-8, and TNF-α by activating the nuclear factor (NF)-κB pathway [7,9]. Besides recruiting immune cells, GFs can regulate the retention and survival of these immune cells via cell–cell interaction molecules such as intercellular adhesion molecule-1 (ICAM-1) [10]. GFs have been shown to inhibit osteoclast differentiation by secreting IL-4 and OPG, highlighting their bone-protective capacity in healthy individuals [5,11]. On the other hand, GFs are capable of inducing osteoclast formation in the presence of oral pathogens like *P. gingivalis*. By inducing RANKL expression and reducing OPG expression, this shifts the balance in favor of osteoclastogenesis, thereby resulting in an increased RANKL/OPG ratio [12]. In general, this crosstalk between GFs and the surrounding cells plays a crucial role in directing the magnitude of inflammatory response in the periodontal tissues [7,13].

In addition, GFs display versatility and plasticity in terms of their potential for differentiating into other cell types, including osteoblast-like cells. It has been shown that GFs that differentiate into osteoblast-like cells highly express osteoblast-related genes [13,14]. In this way, GFs contribute to both aspects of bone homeostasis. This is of biological relevance, as GFs could play a role in regenerative periodontal therapies. As osteogenic differentiation can be achieved by adding ascorbic acid and β-glycerophosphate, fibroblasts have been proposed as efficient models and an alternative source for studying bone-related disorders [14,15].

Thus far, osteogenesis and osteoclastogenesis assays using GFs have evaluated these activities separately. Here, we aimed to investigate the effects of osteogenic differentiation of GFs on their osteoclast-inducing capacity. More specifically, we analyzed how the osteogenic differentiation status, mirrored by the degree of mineralization, affects the ability of GFs to induce osteoclast precursors to differentiate into osteoclasts. To achieve this, GFs were cultured in four groups for 3 weeks without or with osteogenic medium for the final 1, 2, or 3 weeks of step-wise mineralization. To induce osteoclast differentiation, these cultures were then co-cultured for a further 3 weeks with peripheral blood mononuclear cells (PBMCs) containing osteoclast precursors. We hypothesized that the more the GFs differentiated into osteoblast-like cells, the fewer the number of osteoclasts formed would be. Longer exposure to osteogenic medium would thus reduce osteoclast formation.

## 2. Materials and Methods

### 2.1. Cell Preparation

GFs were harvested from twelve systemically healthy donors undergoing their third molar extraction at Vrije University Amsterdam Hospital (Amsterdam UMC, Amsterdam, The Netherlands), from sites with no overt signs of gingival inflammation (probing depth ≤ 3 mm, no bleeding on probing and no clinical attachment loss). Each donor provided written informed consent. Ethical approval exists for transdifferention studies at Vrije Universiteit and were sanctioned by the local Medical Ethical Committee of the Amsterdam University Medical Centers (2016.105).

The free gingiva and partially the interdental gingiva were harvested around the tooth with a scalpel and tissue fragments were washed twice in DMEM (Dulbecco’s minimal essential medium, Gibco BRL, Paisley, Scotland), supplemented with 10% FC1 (Fetal calf serum, HyClone, Logan, UT, USA) and 1% PSF (100 U/mL penicillin, 100 µg/mL streptomycin, and 250 ng/mL amphotericin B [Antibiotic antimycotic solution, Sigma, St. Louis, MO, USA]), chopped into 1 mm fragments, and cultured in a 6-well plate in a humidified atmosphere of 5% CO_2_ in air at 37 °C until GFs reached confluency. Following confluence in a 6-well plate well, cells were transferred to a 75 cm^2^ tissue culture flask (passage 1) and then to two 125 cm^2^ flasks (passage 2). From these flasks, 6 vials were frozen in liquid nitrogen for further experiments (passage 3). Each cell line was established independently for each donor. For all assays, cells of the 5th passage were used.

### 2.2. Osteogenesis

**Figure 1:**
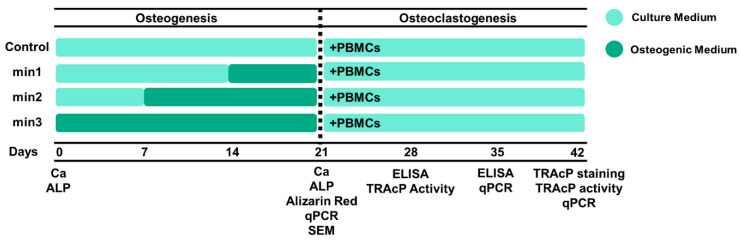
Experimental setup. Gingival fibroblasts (GFs) were cultured in 3 groups for 3 weeks in osteogenic medium for the final 1, 2, or 3 weeks of step-wise mineralization. To induce osteoclast differentiation, these cultures were then co-cultured for a further 3 weeks with peripheral blood mononuclear cells (PBMCs) containing osteoclast precursors. min1, mineralization week 1; min2, mineralization week 2; mineralization week 3; Ca, calcium concentration; ALP, alkaline phosphatase activity; Alizarin Red; Alizarin Red staining; qPCR, quantitative polymerase chain reaction; SEM, scanning electron microscopy; TRAcP, tartrate-resistant acid phosphatase.

GFs were seeded in a 48-well plate (3 × 10^4^ cells/well). The culture medium was composed of DMEM, 10% FC1, and 1% PSF (0.4 mL per well). Cells were cultured in osteogenic medium that contained 10 nM β-glycerophosphate (Sigma, St. Louis, MO, USA) and 50 µg/mL ascorbic acid (Sigma, St. Louis, MO, USA) for 1 (min1), 2 (min2), and 3 (min3) weeks as separate conditions, and only in culture medium (DMEM++) for 3 weeks as control (Figure 1). To assess the mineralization level at week 3, we performed assessments of ALP activity and calcium (Ca^2+^) concentration, DNA measurement, scanning electron microscopy (SEM), Alizarin Red staining, and quantitative PCR (qPCR).

### 2.3. ALP Activity

To assess the ALP activity, cells were harvested at day 0 and week 3 of culturing, lysed in 200 µL water, and stored at −20 °C until the analysis. ALP activity was measured using 4-nitrophenyl phosphate disodium salt (Merck, Darmstadt, Germany) at pH 10.3 as the ALP substrate [16]. Absorbance was read at 405 nm by a Synergy HT spectrophotometer (BioTek Instruments Inc., Winooski, VT, USA). DNA content (in ng/mL) was measured using the CyQuant Cell Proliferation Assay Kit (Thermo Fisher Scientific, Waltham, MA, USA). Fluorescence was measured using a Synergy HT spectrophotometric microplate reader with excitation at 485 nm and emission at 520 nm. ALP activity is shown as ALP/DNA ratio (μmol ALP/ng DNA).

### 2.4. Calcium Concentration

For measuring Ca^2+^ concentration, cells were harvested at day 0 and week 3 of culturing by adding 0.5 mL of 0.5 N acetic acid, and stored at −20 °C until the analysis. For the measurement, 10 μL of sample and 300 μL of working solution (14.8 M ethanolamine/boric acid buffer (pH = 11), orthocresolphtalein complexone (OCPC), hydroxyquinoline (Sigma-Aldrich, St. Louis, MO, USA) in MilliQ (MQ) water) were prepared in duplicate into a 96-well plate and incubated at room temperature for 10 min. Absorbance was read at 570 nm by a Synergy HT spectrophotometer. Ca^2+^ stock solution (183.5 mg calcium chloride dihydrate in MQ water with acetic acid) was used as a standard curve. Ca^2+^ concentration is shown as mg/mL.

### 2.5. Alizarin Red Staining

To analyze mineral deposition, Alizarin Red staining was performed at week 3 after fixation of the cells in 4% formaldehyde for 10 min, rinsing with MQ water. This was followed by the addition of 300 μL of 1% Alizarin Red S solution (Sigma Aldrich) to each well. After incubating for 15 min, cells were washed with MQ water and air-dried. Red nodules were the indicators for mineral deposition.

### 2.6. Scanning Electron Microscopy (SEM)

To visualize the progression of mineralization and cell differentiation at week 3, SEM was used. First, cells were cultured on Thermanox plastic coverslips (Thermo Fisher Scientific), under the same conditions as for tissue culture-treated plastic, followed by a washing step of the coverslips with PBS, and then fixed in 200 μL of McDowell’s fixative. After dehydration using a graded series of ethanol followed by acetone, cells were dried using a critical point dryer (Leica EM CPD300) with liquid carbon dioxide, sputter-coated with platinum–palladium (Leica EM ACE600), then placed on aluminum stubs with carbon stickers and analyzed with a Zeiss Gemini Sigma 300 field emission scanning electron microscope at 5.00 K magnification (EHT = 3.00 kV, WD = 5.5 mm, Signal A = SE2, Carl Zeiss Microscopy GmbH, Jena, Germany).

### 2.7. Osteoclastogenesis

PBMCs were isolated from the buffy coat of a healthy donor who was not involved in the GF isolation procedure (Sanquin, Amsterdam, The Netherlands), using a standard density gradient centrifugation technique with Ficoll-Paque. Permission to use buffy coats to isolate PBMCs for the purpose of osteoclast formation was granted by Sanquin (number: NVT230.0). The buffy coat was diluted 1:1 in 1% PBS citrate (pH 7.4). Afterwards, 25 mL of diluted blood was carefully layered on 15 mL Lymphoprep (Axisshield Po CAS, Oslo, Norway) and centrifuged for 30 min at 800× *g* without braking. The interphase containing the PBMCs was collected, washed three times in 1% PBS citrate, and finally recovered in culture medium. Then, 10^6^ PBMCs were added to the GFs that were cultured for 3 weeks under the conditions described above, and co-cultured for three more weeks to induce osteoclastogenesis [17]. The co-culture medium consisted of DMEM, 10% FC1, and 1% PSF. Osteoclast formation was assessed at different timepoints with qPCR, enzyme-linked immunosorbent assay (ELISA), TRAcP (tartrate-resistant acid phosphatase) activity, and staining.

### 2.8. TRAcP Activity and Staining

To measure the osteoclast formation, TRAcP activity measurement was performed on the cells that were harvested at day 28 and 42. The cells were lysed in 100 μL sodium acetate buffer (0.1 M NaOAc, pH 5.8 + 0.1% Triton X-100) and stored at −20 °C until the analysis. TRAcP activity was measured using 4-nitrophenyl phosphate bis (cyclohexylammonium) salt (Sigma-Aldrich) at pH 5.8 according to the method described by Ljusberg et al. [18]. Absorbance was read at 405 nm by a Synergy HT spectrophotometer. Protein concentration (μg/mL) was measured with the Pierce BCA Protein Assay Kit (Thermo Fisher Scientific) and the absorbance was read at 540 nm. TRAcP activity was shown as arbitrary units per microgram (AU/μg).

After 42 days of culturing, cells were fixed in 4% PBS-buffered formaldehyde for 10 min and washed with H_2_O for TRAcP staining. To identify multinucleated cells, an Acid Phosphatase, Leukocyte (TRAP) Kit (Sigma-Aldrich) was used according to the manufacturer’s instructions. Nuclei were stained with 4′,6-diamidino-2-fenylindool (DAPI) (Thermo Fisher Scientific) for 5 min. Multinucleated TRAcP+ cells were counted using a combination of light and fluorescence microscopy (Leica DMIL and camera DFC7000T, Leica Microsystems, Wetzlar, Germany) from ten standardized images at 100× magnification per well and were considered as osteoclasts when TRAcP+ cells with at least 3 nuclei were present.

### 2.9. ELISA

Supernatants were collected from the co-cultures at day 28 and 35 in order to detect human tumor necrosis factor alpha (TNF-α) levels with ELISA (R&D Systems, Minneapolis, MN, USA) following the manufacturer’s instructions.

### 2.10. qPCR

To extract the RNA from the cultured cells at day 21 and 35, an RNeasy Mini Kit (Qiagen, Hilden, Germany) was used according to the manufacturer’s instructions. The RNA concentration was measured with Synergy (Biotek, Santa Clara, CA, USA). Reverse transcription was performed with the MBI Fermentas cDNA synthesis kit (Vilnius, Lithuania), using both the Oligo(dT)18 and the D(N)6 primers. Real-time PCR primers were designed using the Primer Express software, version 2.0 (Applied Biosystems, Foster City, CA, USA). To avoid amplification of genomic DNA, each amplicon spanned at least one intron.

Real-time PCR was performed on the Light Cycler 480 (Roche, Basel, Switzerland). After an initial activation step of the DNA polymerase for 10 min at 94 °C, 40 cycles were run of a two-step PCR regimen consisting of a denaturation step at 95 °C for 30 sec and an annealing and extension step at 60 °C for 1 min. Subsequently, the PCR products were subjected to a melting curve analysis to test if any unspecific PCR products were generated. The PCR reactions of the different amplicons had equal efficiency. Β_2_-microglobulin was used as the housekeeping gene. The expression of this gene was not affected by the experimental conditions. Samples were normalized for the expression of β_2_-microglobulin by calculating the ΔCt (Ct_gene of interest_ − Ct_Β2_) and the expression of the different genes was expressed as 2^−(ΔCt)^. Primer sequences are listed in Table 1.

### 2.11. Statistical Analysis

Commercial software was used for the statistical analyses (SPSS v. 28.0, IBM, New York, NY, USA) and for the graphs (GraphPad Prism v 10.0.0, La Jolla, CA, USA). Data were not normally distributed (Shapiro–Wilk test) for DNA content, Ca^2+^ concentration, ELISA, qPCR, TRAcP activity, and osteoclast quantification assays; therefore, Friedman’s test with Dunn’s multiple comparisons was performed followed by a Wilcoxon signed rank test for pairwise comparisons between different timepoints. For the normally distributed data (ALP activity), repeated measures ANOVA with Bonferroni correction was performed followed by a paired *t*-test for pairwise comparisons between day 0 and 21. *p*-values ≤ 0.05 were considered statistically significant.

## 3. Results

### 3.1. Osteogenesis

In order to assess the degree of mineralization, GF were cultured either in culture medium (C) or in osteogenic medium for the last 1 week (min1), the last two weeks (min2), or during the whole 3 weeks (min3) of step-wise mineralization.

#### 3.1.1. ALP Activity Is Higher after Three Weeks of Culture

**Figure 2:**
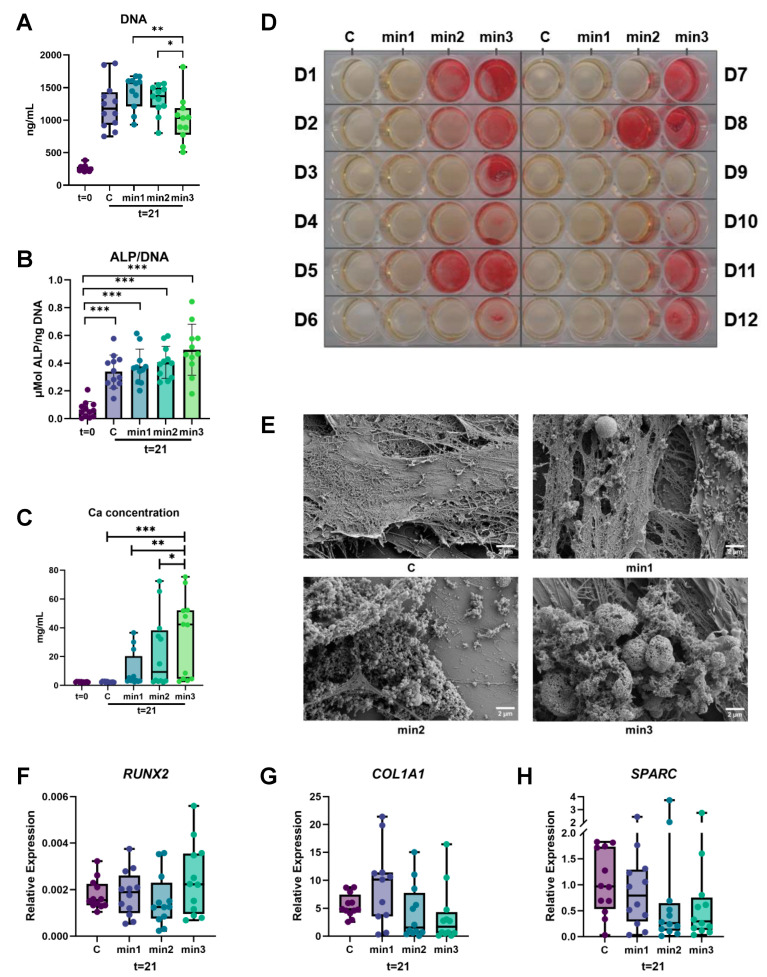
Osteogenic differentiation of GFs. (**A**) Cell numbers increased after 21 days of culture for all conditions. (**B**) ALP activity significantly increased compared to day 0 for all of the conditions. (**C**) Calcium (Ca^2+^) concentration increased over time. (**A**–**C**) The measurements were performed both at day 0 and after 21 days of culturing. (**D**) Alizarin Red staining was performed to visualize the mineral nodules. Red staining represents calcium depositions. D1–D12 represent donors from 1 through 12 (Donor 1–12). (**E**) The mineral nodular-like structures cumulatively increased in number and size from group C to min3, 5.00 K magnification (EHT = 3.00 kV, WD = 5.5 mm, Signal A = SE2). (**F**) *RUNX2* (Runt-related transcription factor 2), (**G**) *COL1A1* (Collagen type I), (**H**) *SPARC* (Osteonectin or Secreted Protein Acidic And Cysteine Rich) expression levels varied between the conditions. (**D**–**H**) The analyses were measured at t = 21. Data are shown as median and range except for ALP activity (mean ± SD). * *p* < 0.05, ** *p* < 0.01, *** *p* < 0.001.

Since ALP activity is always corrected by the number of cells, DNA was measured at day 0 and 21. Cell numbers increased after 21 days of culture for all conditions. Cell number in the min3 condition significantly decreased between min1-min3, and min2-min3 at t = 21 (*p* < 0.01 and *p* < 0.05, respectively) (Figure 2A).

ALP activity was measured both at day 0 and after 21 days of culturing. The enzyme activity significantly increased compared to day 0 for all of the conditions and reached the highest level in min3 where GFs were exposed to the osteogenic medium for the longest period of time (*p* < 0.001). There were no significant differences between the conditions at t = 21, although its level gradually increased within the conditions from C to min3 (Figure 2B).

#### 3.1.2. Higher Calcium Deposition in Cultures That Were Exposed Longer to Osteogenic Medium

To evaluate the Ca^2+^ deposition capacity of osteogenically differentiated fibroblasts, Ca^2+^ was dissolved and assessed both at day 0 and 21. As shown in Figure 2C, Ca^2+^ concentration increased significantly over time and was the highest in min3 compared to the other conditions. The significant differences were found between C-min3, min1-min3, and min2-min3 at t = 21 (*p* < 0.001, *p* < 0.01, and *p* < 0.05, respectively).

This was confirmed with the Alizarin Red staining, another way of visualizing mineral deposition. Figure 2D shows the qualitative assessment of mineral nodules whereby all the donors are represented. Even though there were variations between the donors, there was no mineral deposition observed in the control group and only slight deposition in the min1 group, while the highest mineral deposition was observed in min3 throughout the conditions.

#### 3.1.3. Mineralization Size Increases over Time

SEM images showed that the mineralization process was successfully achieved over time. The mineral nodular-like structures cumulatively increased in number and size from group C to min3 with prolonged exposure to the osteogenic medium as seen in Figure 2E. SEM further revealed that initial mineral deposition (min1) is often on top of the cells rather than on the deposited matrix. Globular mineral deposits are attached more to matrix structures at later stages (min2 and min3).

#### 3.1.4. Expression of Genes Associated with Osteogenesis

The osteogenesis-related gene expression was measured at t = 21 for the genes of early osteogenic marker Runt-related transcription factor 2 (*RUNX2*) (Figure 2F), matrix protein Collagen type I (*COL1A1*) (Figure 2G), and late osteogenic marker Osteonectin (Secreted Protein Acidic And Cysteine Rich (*SPARC*)) (Figure 2H). Although the expression levels of the studied genes varied between the conditions, no statistically significant difference was observed (*p* > 0.05).

In summary, the osteogenesis results show that culturing for up to 3 weeks with osteogenic medium gradually increases osteogenic parameters such as mineral deposition as reflected with calcium deposition assays (calcium measurement and Alizarin Red). SEM analysis further showed an increase in nodular structures over time.

### 3.2. Osteoclastogenesis

#### 3.2.1. Osteogenic Differentiation of GF Inhibits Osteoclast Formation

**Figure 3:**
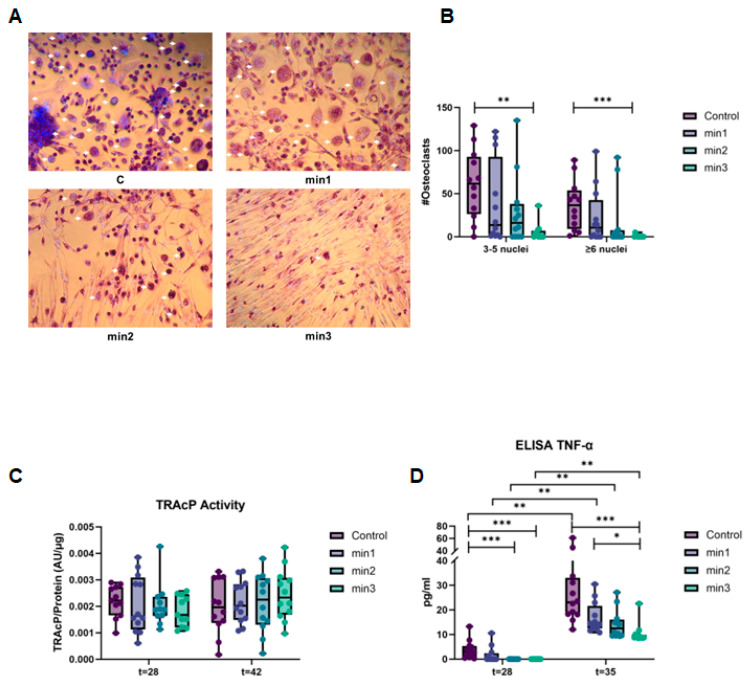
Osteogenic differentiation of GFs inhibits osteoclast formation. (**A**) Micrographs of TRAcP+ multinuclear cells. Cells with 3 or more nuclei, pointed out with white arrows, were counted as osteoclasts. Images were taken at 100× magnification. Blue, DAPI staining for nuclei; purple, TRAcP staining. (**B**) Number of osteoclasts were the highest in groups C and min1 and the lowest in group min3. (**A**,**B**) The quantification and staining were performed at t = 42 (3 weeks of culturing with PBMCs). (**C**) The level of TRAcP activity was similar between conditions at both time points t = 28 (1 week of culturing with PBMCs) and t = 42. (**D**) Overall TNF-α secretion was significantly higher at t = 35 (2 weeks of culturing with PBMCs) than t = 28. At both time points, duration of preceding mineralization caused lower secretion of TNF-α. Data are shown as median and range. * *p* < 0.05, ** *p* < 0.01, *** *p* < 0.001.

Osteoclast formation was determined after co-culturing GFs for a further three weeks with peripheral blood mononuclear cells (PBMCs) that contain osteoclast precursors. The number of TRAcP+ multinuclear cells (Figure 3A) with three or more nuclei was counted at t = 42 (Figure 3B,C). The number of cells with 3–5 or more than 6 nuclei significantly decreased from the control group (C) to min3 (Figure 3B, *p* < 0.01, *p* < 0.001, respectively). Osteoclast formation was the highest in groups C and min1, and the lowest in group min3 where the longest pre-culturing with osteogenic medium was seen (Figure 3B).

TRAcP activity was measured at t = 28 (1 week of culture with PBMCs) and t = 42 (3 weeks of culturing with PBMCs). There were no significant differences between the two time points and between conditions. The level of TRAcP activity was similar between the conditions (Figure 3C).

Since TNF-α can stimulate osteoclast formation even in the absence of RANKL [19], we next assessed whether TNF-α release is influenced by the mineralization regimes. Overall, TNF-α secretion was significantly higher at t = 35 (2 weeks of culturing with PBMCs) than t = 28 (1 week of culturing with PBMCs) (*p* < 0.01). At both time points, longer exposure to osteogenic medium caused lower secretion of TNF-α: there was a significant difference between C-min3 at t = 28 and t = 35 (*p* < 0.001), C-min2 at t = 28 (*p* < 0.001), and min1-min3 at t = 35 (*p* < 0.05) (Figure 3D).

#### 3.2.2. Expression of Genes Associated with Osteoclastogenesis

**Figure 4:**
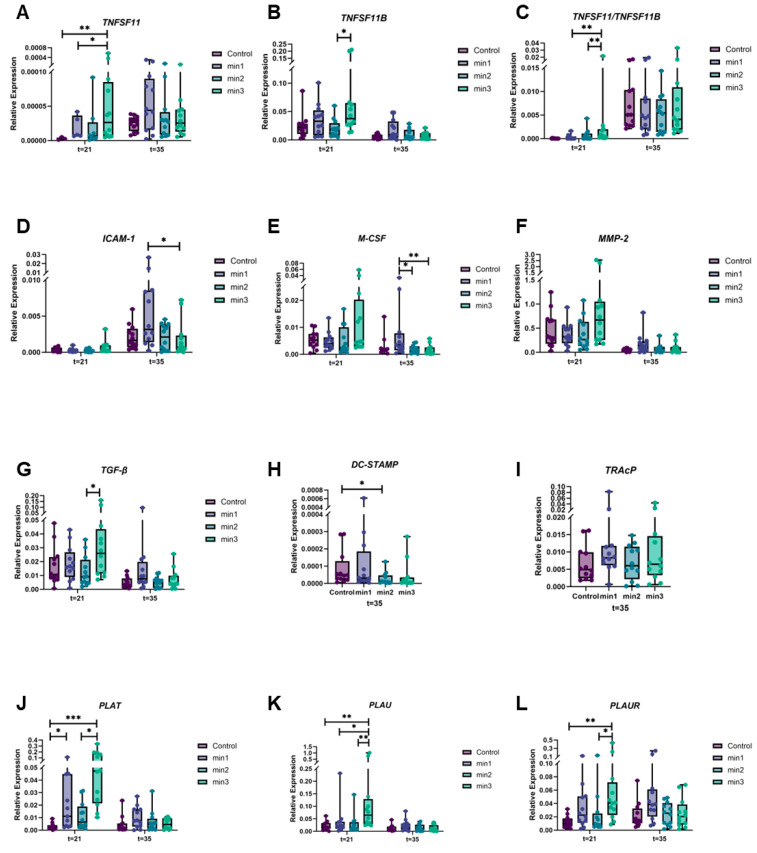
Osteoclastogenesis-related gene expression levels. Gene expression analyses were measured at 21 days and at 35 days for a set of genes that cover the different aspects of the osteoclastogenic capacity such as osteoclast formation, fusion, and cell–cell interactions. The expression levels are shown at both time points; the former (t = 21) addresses only the expression in the monoculture of GFs at the onset of the co-culture, whereas the latter (t = 35) reflects the expression of co-cultures of GFs and PBMCs. (**A**) *TNFSF11* (RANKL); (**B**) *TNFSF11B* (OPG); (**C**) *TNFSF11*/*TNFSF11B* (RANKL/OPG); (**D**) *ICAM-1*; (**E**) *M-CSF*; (**F**) *MMP-2*; (**G**) *TGF-β1*; (**H**) *DC-STAMP*; (**I**) *TRAcP*; (**J**) *PLAT* (t-PA); (**K**) *PLAU* (u-PA); (**L**) *PLAUR* (u-PA). Data are shown as median and range. * *p* < 0.05, ** *p* < 0.01, *** *p* < 0.001.

Osteoclastogenesis-related gene expression was measured at day 21 and day 35 (14 days after the addition of PBMCs), for a set of genes that cover the different aspects of the osteoclastogenic capacity such as osteoclast formation, fusion, and cell–cell interactions. The expression levels are shown at both time points; where the former (t = 21) addresses only the expression in the monoculture of GFs at the onset of the co-culture, the latter (t = 35) reflects the expression of co-cultures of GFs and PBMCs (Figure 4).

RANKL (*TNFSF11*; Figure 4A) expression was significantly higher at min3 than C and min1 at t = 21 (*p* < 0.01 and *p* < 0.05, respectively), indicating that the longer exposure to osteogenic medium increased RANKL expression. No significant difference was observed between the conditions at t = 35. OPG (*TNFSF11B*; Figure 4B) levels were the highest at min3 and significantly higher than min2 at t = 21 (*p* < 0.05). Overall, OPG levels were several folds higher than RANKL at both time points, hence, the RANKL/OPG ratio (*TNFSF11*/*TNFSF11B*; Figure 4C) was comparable throughout the conditions at t = 35 but significantly higher at min3 compared to min1 and min2 at t = 21 (*p* < 0.01 and *p* < 0.01, respectively).

Another important molecule for osteoclast formation is ICAM-1 that is required for cell–cell interaction between GF and PBMCs. ICAM-1 expression levels were significantly higher at min1 compared to min3 at t = 35 (*p* < 0.05; Figure 4D). M-CSF expression, crucial for the proliferation of osteoclast precursors, was the highest at min1 and significantly higher than min2 and min3 at t = 35 (*p* < 0.05 and *p* < 0.01, respectively; Figure 4E). MMPs are important for osteoclast migration by controlling the cell–matrix interactions and solubilizing bone matrix [20]. However, MMP-2 expression levels showed no significant differences between the conditions at any time points (*p* > 0.05; Figure 4F). TGF-β may substitute RANKL and blocking of its signaling route inhibits osteoclast formation [19,21]. TGF-β expression levels were significantly higher at min3 than min2 at t = 21 (*p* < 0.05; Figure 4G). DC-STAMP is considered as the gene of importance in cell–cell fusion and there was a significant difference between C and min2 in DC-STAMP expression at t = 35 (*p* < 0.05; Figure 4H). TRAcP expression levels, a marker of osteoclast activity, were similar throughout the conditions at t = 35 (*p* > 0.05; Figure 4I).

Fibrinolytic factors such as t-PA, u-PA, and its receptor, u-PAR have roles in the regulation of bone metabolism process. These factors participate in the differentiation of osteoclast precursors into osteoclasts, modulate the induction ability of osteoblasts on osteoclast differentiation, and cause osteoblasts to mineralize the ECM. The changes in these factors affect the functions of both osteoblasts and osteoclasts [22,23,24]. For this reason, we investigated the gene expression levels of t-PA, u-PA, and its receptor, u-PAR. t-PA (*PLAT*; Figure 4J) expression was its highest at min3 and significantly higher compared to C and min2 at t = 21 (*p* < 0.001 and *p* < 0.05, respectively). There was also a significant difference between C and min1 at t = 21 (*p* < 0.05). Similar to t-PA, u-PA (*PLAU*; Figure 4K) reached its highest expression levels at min3 and was significantly higher than C, min1, and min2 at t = 21 (*p* < 0.01, *p* < 0.05, and *p* < 0.01, respectively). Significantly higher expression was observed in u-PAR, the receptor of u-PA (*PLAUR*; Figure 4L), in min3 compared to C and min2 at t = 21 (*p* < 0.01 and *p* < 0.05, respectively).

## 4. Discussion

This study is the first to describe the effects of osteogenic differentiation, here of human GFs, on osteoclast-formation-inducing capacity. Here, we first of all showed that GFs are capable of acquiring an osteogenic phenotype in the presence of osteogenic stimuli. Secondly, the GFs that were cultured longest in osteogenic medium reduced their osteoclast-inducing capacity.

The main characteristics of GFs are to support ECM production and maintenance and to respond to cytokines and physical signals from the surrounding matrix and cells. Once activated, they modify cell recruitment and proliferation, and participate in different physiological and pathophysiological processes such as wound healing and chronic inflammation [25]. Additionally, due to sharing major similarities with MSCs in terms of their plasticity and ability to adopt different lineages under various external stimuli, GFs can be considered as a good source for tissue regeneration [14,25].

ALP is a requisite enzyme for mineralization processes by supplying inorganic phosphate for bone matrix maturation. In that sense, it is one of the mediators of osteogenic differentiation [17]. When GFs are cultured in the presence of osteogenic medium, we observed that ALP activity and Ca^2+^ deposition increased over time [26]. These findings, together with the calcium deposition as assessed by the dissolved calcium measurement, the Alizarin Red staining, and SEM images, indicate that GFs exhibited an osteogenic phenotype under the appropriate experimental conditions. The increase in mineralization was shown to be time-dependent, with gradual increases from 1 to 3 weeks of mineralization.

Interestingly, osteogenesis-related gene expression levels of *RUNX2*, *COL1A1*, and *SPARC* were not statistically different. *RUNX2*, a master gene of osteoblast differentiation, demonstrated similar expression levels throughout the conditions, although a slight increase was observed at min3. It has been shown in the literature that *RUNX2* has essential roles in the upregulation of *ALP*, *COL1A1*, and *SPARC*, and the overall expression of these genes is considered as an indication for the proliferation phase of osteoblast progenitors accompanied by ALP activity [26]. In a different approach, it has been shown that GFs, incubated in osteogenic medium, significantly downregulated *RUNX2* at days 14 and 21, but strikingly peaked in *SP7*/*OSX* expression at day 14 [27]. In contrast to the findings of these studies, *RUNX2*, *COL1A1*, and *SPARC* levels did not show any significant differences in this study; variations in donors and heterogeneity of cell populations may account for this discrepancy. Bruderer et al. reported that *RUNX2* positively regulates *OPG* gene expression, hence, inhibiting osteoclast formation, indicating the molecular connections between osteogenesis and osteoclastogenesis [28]. This may correlate with higher expression levels of the genes mentioned above for min3 in the present study. One of the inevitable limitations of the current study is that, due to the treatment regime, only 3 weeks of culture could be assessed. *RUNX2* and *ALP* expression typically peak earlier during osteogenic stimulation [26].

Osteoclasts are terminally differentiated multinucleated cells that originate from monocytes under the influence of several factors. Once differentiated, they resorb bone via secretion of acid and proteolytic enzymes such as Cathepsin K and TRAcP [29]. TRAcP is considered as an important marker for osteoclast activity but is also expressed by mononuclear osteoclast precursors [30]. It is also suggested that TRAcP is secreted by osteoclasts regardless of whether they are resorbing [30]. In the present study, TRAcP activity and gene expression levels were stable throughout the conditions at all time points even though the number of the osteoclasts dramatically decreased over time. These findings may be explained by the fact that TRAcP can also be expressed by mononuclear cells which were mostly seen in the conditions at the further stages of mineralization.

RANKL and M-CSF are regarded as critical cytokines for osteoclast formation, where M-CSF is important for the proliferation of osteoclast precursors and RANKL an important differentiation factor. OPG is the decoy receptor for RANKL and prevents RANKL/RANK interaction [4,26]. In the present study, *RANKL* and *OPG* expression and *RANKL*/*OPG* ratio were the highest in min3 compared to the other conditions at t = 21, and *M-CSF* levels were significantly higher at min1 than min2 and min3 at t = 35. With higher expression of the osteoclastogenesis genes at the beginning of co-culture, it therefore seems paradoxical that three weeks of mineralization leads to lower osteoclast formation. It has also been shown that M-CSF mostly modulates the resorbing activity of the osteoclasts rather than their survival in the later stages of osteoclastogenesis [31]. In this context, the observed results may indicate that longer exposure to osteogenic medium and the maturation stage affects the osteoclast-forming capacity of the GFs. Although initially *RANKL* was the highest in the min3 condition at 21 days, this was no longer seen during the co-culture period.

TNF-α exerts its effects on osteoclastogenesis by stimulating the osteoclast formation via increasing *RANKL* expression, potentiating the effects of IL-1, and stimulating M-CSF production [32]. Importantly, as confirmed here and as originally described by de Vries et al. and reviewed elsewhere, *OPG* is at least 100-fold higher than *RANKL* in this culture system [5,33]. TNF-α has been described as an additional osteoclastogenic factor that can make RANK^−/−^ precursors differentiate into osteoclasts independent of the RANKL/RANK/OPG axis [19]. Overall TNF-α protein levels were found to be elevated at t = 35 compared to t = 21, and higher in control conditions compared to the 3 weeks of mineralization condition, which parallels the decrease in osteoclast formation.

Osteoclast precursors express osteoclastic and monocytic markers depending on their differentiation stage [34]. ICAM-1 is required for cell–cell interaction between osteoclast precursors and either osteoblasts, fibroblasts, or endothelial cells, and also for preosteoclast extravasation and osteoclast formation [34,35]. It has been shown that ICAM-1 on osteoblast-like cells interacts with the corresponding binding receptor LFA-1 on osteoclast precursors in the beginning of osteoclast formation and causes upregulation of *M-CSF*, *TNF-α*, and *RANKL* [35,36]. The ICAM-1 results in this study showed the lowest expression levels in min3 at t = 35, suggesting that ICAM-1 participates in the early stages of the osteoclastogenesis.

MMPs are involved in several biological processes, one of them being bone metabolism [20]. They solubilize collagen for osteoclast migration by controlling the cell–matrix interactions required for cell attachment as well as for the viability and functionality of osteoblasts, osteoclasts, and osteocytes [20,37]. It has been shown that MMP-2 in particular has direct effects on osteopontin that promotes osteoclast activity and on bone sialoprotein which is involved in osteoblast development. MMP-2 also alters osteoblast and osteoclast development by causing the release of TGF-β from degraded bone matrix [37]. In this study, *MMP-2* levels were high at min3 at t = 21. TGF-β is proposed as a stimulating factor for bone formation through the recruitment, proliferation, and differentiation of osteoblasts and as an inhibitor of osteoclast bone resorption [29,32]. As shown by Ota et al., TGF-β stimulates osteoclast-derived Wnt10b production that may contribute to bone formation [38]. The increased *TGF-β* levels in min3 at t = 21 that were observed in this study are consistent with the literature on this aspect.

Osteoclast differentiation comprises a complex cascade of events that directs osteoclast precursors to become fully functional bone-degrading multinucleated cells [4]. DC-STAMP is one of the crucial regulators of cell fusion [39]. Yagi et al. showed that osteoclasts isolated from DC-STAMP knockout mice were mononucleated due to the deficiency in cell fusion mechanisms [40]. *DC-STAMP* levels were found to be decreased over time of preceding mineralization which correlates with the lower number of osteoclast and more mononucleated cells observed in this study [39].

It has been shown that plasmin activates TGF-β in the bone matrix leading to increased *OPG* expression and eventually suppression of osteoblast-mediated osteoclastogenesis [41]. Like plasmin, other fibrinolytic factors such as t-PA, u-PA, and its receptor u-PAR are produced by osteoblasts and osteoclasts [42]. It has been observed that proteolytic activation of TGF-β that is controlled by fibrinolytic factors influences bone formation by stimulating the recruitment, proliferation, and differentiation of osteoblast-like cells [43]. Daci et al. showed that a lack of t-PA and u-PA in mice resulted in an increased bone mass and accumulation of bone matrix proteins, suggesting an indirect role of fibrinolytic factors in osteoblast function and bone formation [22]. It is therefore surprising that our study showed the highest *t-PA* but also *u-PA* and *u-PAR* at 3 weeks with osteogenic medium, suggesting a relation between osteogenesis and increased fibrinolytic factors. The same group also reported that *t-PA*, *u-PA*, and *PAI-1* expression is not required for osteoclast formation nor a number of osteoclasts, but rather for the degradation of non-collagenous components of the bone-like matrix [44]. In addition to this, Everts et al. reported that bone degradation by osteoclasts was delayed due to the lack of fibrinolytic factors and these factors are involved in the early phases of bone resorption via the integrin-mediated attachment of osteoclasts to the bone surface [23]. In another study, when co-cultured with osteoblasts from Plg^−/−^ mice, plasminogen/plasmin deficiency resulted in lower *OPG* expression levels from osteoblasts but similar ALP activity, more TRAcP+ multinucleated cells, and upregulation of bone resorption activity from osteoclasts, suggesting that plasmin attenuates osteoclastogenesis and clearly revealing that fibrinolytic factors regulate both osteoblast and osteoclast functions, as also shown by research from Sulniute et al. where plasmin deficiency resulted in persistent inflammation, excessive neutrophil accumulation, and severe alveolar bone loss [24,45].

In order to explain the effects of increased mineralization on a decreased osteoclast formation, several aspects of the cell–matrix interactions should be considered. The ECM microenvironment, composed of several specific components, regulates not only matrix mineralization and maturation processes, but also cell differentiation [46]. Hwang et al. showed that ECM stiffness and crosslinking influence osteoblast and osteoclast behavior [47]. Osteoblast differentiation and maturation were increased in a dense matrix as seen by increased ALP protein levels, mRNA *RUNX2*, *ALP*, and osteocalcin (*OCN*) levels. ECM crosslinking increased osteoblast-mediated mineralization in a dense matrix compared to a soft matrix as assessed by Alizarin Red S staining and calcium deposition measurement. In contrast to osteoblasts, osteoclast formation occurred more in a soft matrix than in a dense matrix [47]. ECM crosslinking affected the number and size of osteoclasts where larger osteoclasts were observed on a soft matrix. Likewise, osteoclast maturation, as illustrated by TRAcP staining and osteoclast number, was the highest on the soft matrix and decreased with increasing ECM crosslinking. They also concluded that osteoblasts cultured on a soft matrix are more likely to promote osteoclastogenesis, whereas those cultured on a dense matrix are less likely to do so. In other words, a dense matrix would give rise to osteoblasts just as a soft matrix would give rise to osteoclasts. Taken together, one explanation for the observed results in this study may be the effects of matrix maturation over time. When the mineral is layered on top of the GFs, such as seen in SEM, the mineralized matrix may prevent cell–cell mediated differentiation, resulting in less osteoclast formation. Another reason could be that, similar to the study mentioned above, matrix stiffness eventually alters cell behavior, where the matrix in the early stages of osteogenic differentiation consisted mainly of type I collagen, leading to more osteoclast formation, whereas as differentiation progressed, a more dense mineralized matrix inhibited osteoclast formation.

Regarding possible limitations of the present study, the variations between donors should be mentioned. Being aware of this aspect, we therefore included no less than 12 donors. Such analyses like qPCR should be also performed not only at the end of the osteogenesis but also at additional interim time points that may help monitor the osteogenesis part in more detail. The lack of resorption assay to assess the osteoclast function may be considered as another limitation. Overall, the results clearly show that GFs are capable of acquiring an osteogenic phenotype, and this results in reduced osteoclast formation.

## 5. Conclusions

GFs have been shown to differentiate into osteoblast-like cells that deposit calcium in the form of nodules. Their degree of differentiation reduces their osteoclast-inducing capacity. This further indicates that, with appropriate stimulation, GFs have the potential to be used in regenerative periodontal treatments. Therefore, future research could help establish the exact mechanisms and pathways that regulate differentiation processes and their effects on osteoclast formation.

## Figures and Tables

**Table 1:** Primer sequences used for quantitative RT-PCR.

Gene	Sequence 5′-3′	Amplicon Length (bp)	Ensembl Gene ID
*β_2_M*	AAgATTCAggTTTACTCACgTC	293	ENSG00000166710
	TgATgCTgCTTACATgTCTCg		
**Osteogenesis**			
*RUNX2*	ATgCTTCATCgCCTCAC	156	ENSG00000124813
	ACTgCTTgCAgCCTTAAAT		
*COL1A1*	TCCAACgAgATCgAgATCC	190	ENSG00000108821
	AAgCCgAATTCCTggTCT		
*SPARC*	TACATCgggCCTTgCAAATAC	100	ENST00000231061
	AgggTgACCAggACgTTCTTg		
**Osteoclastogenesis**			
*TNFSF11*	CATCCCATCTggTTCCCATAA	60	ENSG00000120659
	gCCCAACCCCgATCATg		
*TNFRSF11B*	CTgCgCgCTCgTgTTTC	100	ENSG00000164761
	ACAgCTgATgAgAggTTTCTTCgT		
*ICAM1*	TgAgCAATgTgCAAgAAgATAgC	104	ENSG00000090339
	CCCgTTCTggAgTCCAgTACA		
*CSF1*	CCgAggAggTgTCggAgTAC	100	ENSG00000184371
	AATTTggCACgAggTCTCCAT		
*MMP2*			
*TGFB1*	CACCCgCgTgCTAATggT		
	CTCggAgCTCTgATgTgTTgAA		
*DCSTAMP*	ATTTTCTCAgTgAgCAAgCAgTTTC	101	ENSG0000016493
	AgAATCATggATAATATCTTgAgTTCCTT		
*ACP5*	CACAATCTgCAgTACCTgCAAgAT	128	ENSG00000102575
	CCCATAgTggAAgCgCAgATA		
**Fibrinolysis**			
*PLAT*	CggACTggACggAgTgTgA	104	ENSG00000104368
	TggATgggTACAgTCTgACATgA		
*PLAU*	TggAACTCTgCCACTgTCCTT	100	ENSG00000122861
	TTgTCTgggTTCCTgCAgTAATT		
*PLAUR*	ATCggACTggCTTgAAgATCA	101	ENSG00000011422
	ggCTTCgggAATAggTgACA		

*β2M*, β2-microglobulin; *RUNX2*, runt-related transcription factor 2; *COL1A1*, collagen type 1A; *SPARC*, osteonectin; *TNFSF11*, tumor necrosis factor superfamily member 11 (coding for receptor activator of nuclear factor-κB ligand (RANKL)); *TNFRSF11B*, tumor necrosis factor receptor superfamily member 11b (coding for osteoprotegerin (OPG)); *ICAM1*, intercellular adhesion molecule 1; *CFS1*, colony-stimulating factor-1 (coding for macrophage colony-stimulating factor (M-CSF)); *MMP2*, matrix metalloproteinase-2; *TGFB1*, transforming growth factor beta-1; *DCSTAMP*, dendritic cell-specific transmembrane protein; *ACP5,* acid phosphatase 5, tartrate resistant (coding for tartrate-resistant acid phosphatase (TRAcP)); *PLAT*, plasminogen activator, tissue type (coding for tissue-type plasminogen activator (t-PA)); *PLAU*, plasminogen activator, urokinase (coding for urokinase-type plasminogen activator (u-PA)); *PLAUR*, plasminogen activator, urokinase receptor (coding for urokinase receptor (u-PAR)). For each gene, the first sequence represents the forward primer, and the second sequence the reverse primer.

## Data Availability

All relevant data from this study are included in the article. Further inquiries can be directed to the corresponding author.
